# Lower Leg Injury Mechanism Investigation During an IED Blast Under a Vehicle Using an Anatomic Leg Model

**DOI:** 10.3389/fbioe.2021.725006

**Published:** 2021-11-17

**Authors:** Sławomir Suchoń, Michał Burkacki, Kamil Joszko, Bożena Gzik-Zroska, Wojciech Wolański, Grzegorz Sławiński, João Manuel R. S. Tavares, Marek Gzik

**Affiliations:** ^1^ Department of Biomechatronics, Faculty of Biomedical Engineering, Silesian University of Technology, Zabrze, Poland; ^2^ Department of Biomaterials and Medical Devices Engineering, Faculty of Biomedical Engineering, Silesian University of Technology, Zabrze, Poland; ^3^ Faculty of Mechanical Engineering, Institute of Mechanics and Computational Engineering, Military University of Technology, Warszawa, Poland; ^4^ Departamento de Engenharia Mecânica, Faculdade de Engenharia, Instituto de Ciência e Inovação em Engenharia Mecânica e Engenharia Industrial, Universidade do Porto, Porto, Portugal

**Keywords:** armoured vehicle, explosion, injury mechanism, tibia fracture, deck-slap fracture, trauma biomechanics, lower limb biomechanics, blast injury

## Abstract

Attacks with improvised explosive device (IED) constituted the main threat to, for example, Polish soldiers in Iraq and Afghanistan. Improving safety during transport in an armored vehicle has become an important issue. The main purpose of the presented research is to investigate the mechanism of lower leg injuries during explosion under an armored vehicle. Using a numerical anatomic model of the lower leg, the analysis of the leg position was carried out. In all presented positions, the stress limit of 160 (MPa) was reached, which indicates bone damage. There is a difference in stress distribution in anatomic elements pointing to different injury mechanisms.

## Introduction

Over the years, there has been an increased interest in the problem of protecting soldiers during transport, against the risks resulting from an explosion of a mine or improvised explosive device (IED). The problem has become the subject of interest of researchers in the various fields of medicine, technology, and defense. As an example of early research, the work of (Radonic et al., 2004) can be cited. His team analyzed the effects of mine explosions in the Balkan conflict in the 1990s from a medical point of view.

Efforts were made to a better understanding of the mechanisms of the impact of the explosion on the vehicle and passengers to search for ways to counteract their effects. The culmination of these activities resulted in the introduction of vehicles with increased mine protection in Iraq and Afghanistan by coalition forces, in particular by the US forces with the purpose of counteracting an increasing number of IED attacks.

IED attacks were the main cause of losses among Polish soldiers in Iraq and Afghanistan. Statistics on Polish soldiers indicate that the IEDs are the cause of 47% of losses in Iraq and 63% of losses in Afghanistan (iCasualties, 2017).

One of the measures to increase the safety of soldiers in transport or during patrol is the introduction of Mine-Resistant Ambush-Protected (MRAP) vehicles. These are vehicles with features that, to some extent, make them resistant to the effects of an explosion under the hull. These features are mainly a high suspension distancing the hull away from the explosive charge and a V- or U-profiled bottom of the hull, which helps dissipate energy and reduce the impact of the shock wave. Due to their specialized function, MRAP vehicles have a few disadvantages. They have a high body and a high center of gravity, which makes them more susceptible to tipping over as a result of an explosion or a road accident, or when overcoming obstacles. This is such a serious problem that US command has introduced roll simulation to the training of soldiers (TR-HFM-148, 2012).

**FIGURE 1:**
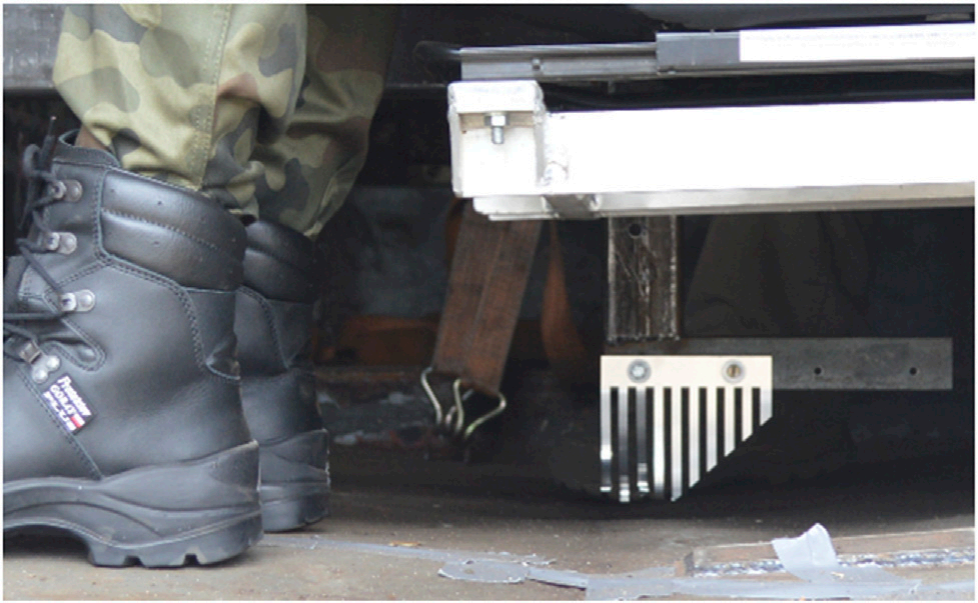
Elastic deformation of the floor (4 cm) recorded with a measuring comb after the explosion of a 1.7-kg TNT charge under the vehicle.

The process of an IED explosion under the vehicle is very violent and turbulent. The explosion creates a number of threats to the safety of the crew and equipment (TR-HFM-149). The deformation of the structure occurring as a result of the explosion is the most important effect from the point of view of a lower limb safety. During the explosion, there are plastic and elastic deformations (Figure 1) (called local effects) that transfer dynamic impact on the crew, especially on their lower limbs. Local effects are the dominant phenomenon that causes injuries within about 0.5–1 ms from the time of detonation. In opposition, dismounted soldiers are more prone to shock wave, debris, and shrapnels, which can cause devastating damage to soft tissues and bones (Rankin et al., 2020). After that, the entire vehicle body begins to accelerate due to the impact of the explosion wave.

The effects of moving the entire vehicle are called global effects. They become the dominant phenomenon occurring within about 15 ms of the detonation. Local and global effects affect the vehicle and crew in the first phase of the explosion. When the vehicle descends and hits the ground, there is another threat in the form of an acceleration pulse with the opposite direction to the acceleration from the explosion. After hitting the ground, there are further effects of the explosion, which include rolling, collision, or overturning of the vehicle, which are comparable to traffic accidents. The mechanism of the impact of the explosion on the crew and the threats posed by the explosion under the vehicle are reflected in the epidemiology of crew injuries.

Analysis carried out by Ramasay et al. (2011a) distinguishes cases of IED explosions under the vehicle, where the map of skeletal injuries is as follows: 3.3% humerus, 1.7% radial or elbow bone, 3.4% hand bones, 10.1% femur, 45.8% tibia or fibula, and 35.6% of the foot bone.

Both *in vivo* and *in vitro* lower limb injuries resulting from the explosion of mines or IEDs are quite well documented in the literature (Kuppa et al., 2001; Nelson et al., 2008; Nguyen et al., 2020; Radonic et al., 2004; Ramasay et al., 2013). These injuries are dislocations and sprains of the ankle (AIS1), patella injuries and ligament injuries in the knee joint (AIS 2), fractures of the foot bones, in particular the talus and calcaneus (AIS2), fibula and tibia fractures, including the fracture of the proximal tibia (AIS2+), the fracture of the femur (AIS2+), and pelvic fractures (AIS2+).

Vehicle safety assessment is carried out according to the NATO Standardization Agreement “Protection Levels for Occupants of Logistic and Light Armored Vehicles”. This document includes an assessment method for threats coming from mine blasts, IED blasts, and a kinetic energy impulse. Testing methodology indicates *inter alia* the type of an Anthropometric Test Device—ATD (human surrogate) and injury criteria determining the risk of injuries. Depending on the blast scenario, the ATD that is selected is either EuroSid-2re (for side impacts) or Hybrid-III 50th male dummy with the Mil-LX lower limb (in the case of other scenarios). Post mortem human surrogates are rarely used only for research purposes. Injury criteria are a set of parameters correlated with probability and severity of injury. Severity of injury is classified by the Abbreviated Injury Scale (AIS). The adopted methodology is designed for the vehicle assessment; therefore, it is heavily quantified and, at some point, very general. This is why specified research work has to be done to investigate specific injuries or its mechanics. Numerical simulation seems to be a well-adopted tool for this purpose.

## Materials and Methods

The anatomical model of the lower limb, which will allow to carry out an extensive analysis and explain the presented issues should map the anatomical structure of the skeletal system of the human lower limb; enable the analysis of phenomena occurring in bones, and thus take into account their mechanical properties; take into account the soldier’s boot; enable the results obtained to be referenced to experimental or numerical studies using the H-III dummy; and be relatively easy to use as a tool in the research.

Commercial ATD models, including H-III, do not fully cover these needs to provide complete information about the mechanism of injury such as the type of fracture or its location. Commercial anatomical models, even models developed for automotive, are not adapted to high dynamics occurring during explosion events. In this case, the only way is to use proprietary models that are dedicated to specific issues.

To tackle the abovementioned tasks, it is necessary to develop a model that will allow the performance of tests on the scale of the lower limb itself in conditions of an explosion under the vehicle.

In the study, it used a model of a single soldier with an anatomical lower limb. The boundary conditions used in simulation were adopted from a related model of vehicle with six HIII dummies presented by Gzik et al. (2017).

It was decided to use the Madymo software to build the proposed model. It is a tool widely used in the automotive industry, among others by Honda, Fiat, and Hyundai. The Madymo was also used for military purposes to conduct dynamic analyses of the effects of an explosion under a vehicle or emergency helicopter landing by Vadlamudi et al. (2011).

Methodology with a similar approach can be found in Rebelo et al. (2021), which presents FEM model of lower limb used for evaluation of blast mitigation systems and evaluates protection offered by a combat boot.

### Geometry and Material Properties

The simplified assumptions were made based on the literature review including anatomical models of the lower limb. Assumptions were determined as follows:

• anatomical structure of the model has been made only for the lower leg and foot area;
• due to the load mechanism in the event of an explosion below the vehicle, soft tissues are ignored in the load transfer process;
• limb model should enable the replacement of the H-III dummy limb;
• material heterogeneity of the bone was taken into account and divided into cortical and spongy parts; and
• due to the complicated anatomical structure of the foot:
  o bone of the lower leg and proximal walk (tibia, fibula, calcaneus, and talus) are modeled as deformable elements, and the remaining foot bones will be modeled as non-deformable elements;
  o joints created by the distal tarsus, metatarsus, and forefoot are treated as fixed joints.

The geometric model was obtained from medically computed tomography images processed in the Mimics and 3-matic package. The bone surfaces were smoothed. The output files were surface models used in further work to generate the simulation mesh. It was decided to use hexa8 hexagonal elements. Discretization using hexagonal elements is relatively difficult and time-consuming at the same time, and it requires simplifications in geometry. On the other hand, it allows the researchers to get good quality results at a relatively good calculation cost. These are big advantages of dynamic simulations.

**FIGURE 2:**
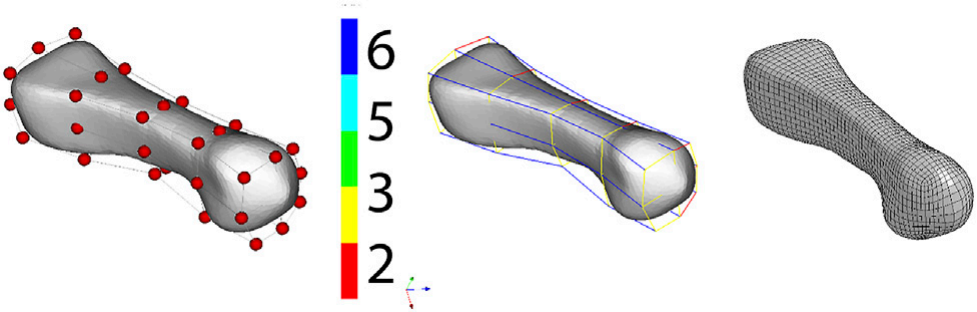
Process of creating a hexa mesh in the IA-FEMesh software illustrated by the example of metatarsal bone.

The volumetric hexa mesh was created on the basis of the obtained surface geometries. The IA-FEMesh software was used for this purpose. A critical point in the discretization process is to describe the surface of the geometry using blocks and to determine the number of elements that make up the block, which translates into the density of the mesh (Figure 2). The program allows the division of mesh elements into material groups, which were further divided into cortical and trabecular parts. It should be noted that obtaining a hexa mesh for anatomical shapes was a compromise between the simplification of the input geometry and the quality of mesh elements. Parameters describing the mesh quality of individual bones are presented in Table 1.

**TABLE 1:** Material parameters selected for individual model elements.

Model element	Material	*E* (GPa)	*ν*	*ρ* (kg/m^3^)	Yield stress (MPa)
Cortical part of tibia	Cortical bone	21.4	0.3	1,800	160
Trabecular part of tibia	Trabecular bone	10.6	0.225	2,000	100
Others (including fibula, talus, calcaneus)	Homogeneous bone	10	0.34	2,000	160

Files with a volumetric mesh were prepared as k-files in the LS-PrePost pre-processing program and then imported as xml files into the Madymo environment.

An isotropic elastic–plastic material model (MATERIAL.ISOPLA built-in Madymo model) was used to reproduce bone properties. The material deforms elastically in accordance with Hooke’s law, until it reaches the value of stress determined by the yield point, after which it exhibits a plastic character. Exceeding the yield point will indicate bone damage. The model properties were selected based on literature research. Table 2 presents the values of material parameters of individual model elements. The division into cortical and spongy bone was used in the tibia by prescribing appropriate values. The other lower limb bones were assigned homogeneous material.

**TABLE 2:** Additional mass assigned to elements.

Limb fragment	H-III [kg]	FE elements of skeleton [kg]	Soft tissues mass assigned to elements [kg]
Upper lower leg	1.699	0.346	1.352
Lower leg	1.930	0.346	1.584
Foot (tarsus and -metatarsus)	1.229	0.254	0.975
Forefoot	0.164	0.08	0.087
Phalanges	0.084	0.02	0.057
Total	5.109	1.054	4.055

Reaching the stress value at the yield strength level is a premise for tissue damage, making the stress value become, in a sense, a traumatic criterion. However, it should be noted that when observing an injury, attention should also be paid to the number of elements where the value is reached and the shape of the stress field. Depending on the number of elements covered by this value and the shape of the stress field, it can only be deduced if, for example, the bone was cracked and not fractured. This is not a sharp, quantified criterion, but on the other hand, this approach provides much more information about the site of the highest limb load values or load transfer mechanisms, as opposed to classic-approach criteria, e.g., a compressive force criterion in the lower tibia.

### Model Structure

The first stage in the construction of the hybrid model was the preparation of the H-III dummy for limb modification. For this purpose, the dummy’s knee was replaced with a model of a femoral fragment—a condyle with a piece of shaft. This extension of the dummy’s leg tubular fragment with a femoral fragment forms a kind of interface for the anatomical part of the model. The borderline between the dummy model and the anatomical model of the lower limb is the femur bone body element terminating the kinematic chain from the dummy and starting the kinematic chain towards the lower limb. The combination of cylindrical and sliding joints allows the reproduction of anatomical rotational-translational motion in the knee joint. The range of motion and resistance, i.e., the moment of force needed to move depending on the change of position in the joint, are determined by the knee-joint characteristics in the axis of rotation and in the direction of knee movement. The characteristics used are the same as for the dummy joint.

The first element of the kinematic chain of the lower limb is the tibia element connected to the knee joints and ankle joint. The connection uses only nodes lying on the articular surface of bones. The same was done with the fibula bone, which is connected by means of subosseous membranes with the tibia.

The upper part of the ankle joint is formed by the spherical joint. The characteristics of this connection are in line with those of the dummy’s ankle. The joint connects the tibia element with the talus element to which the finite element nodes have been assigned, forming the articular surface of the upper ankle joint (between the talus, tibia, and fibula).

The lower ankle joint, formed by the talus and calcaneus, is the connection of the talus element with the nodes assigned to the calcaneus *via* the joint that ensures anatomically correct mobility (movement of inversion and eversion of the foot), and allows compression of the joint under axial load on the limb. The relationship between the talus and the calcaneus bone was defined by the contact between finite elements. The distal tarsus bones are firmly connected with ligaments and their joints are motionless or have little mobility, so it was decided to combine these bones into one group. The medial, intermediate, and lateral cuneiform bones, as well as the navicular and cuboid bones, treated as non-deformable FE elements, were assigned to one element, which is connected with a cylindrical joint to the calcaneus body element to form the Chopart joint. This joint also has minimal mobility as the only degree of freedom of this joint has been blocked. The use of the abovementioned joint instead of a rigid joint is justified as it creates the possibility of positioning the foot at different stages of various simulation scenarios. An analogous procedure was adopted in the case of metatarsal bones, which were also assigned to one element connected to a stiffened joint.

Analogical operations were made for the first, second, and third line of phalanges. The joints of the phalanges were not stiffened, but they were assigned stiffness characteristics like the toes of the dummy’s foot.

The shoe model was taken as a built-in model from Madymo. It consists of FE surface elements whose characteristics were determined experimentally and implemented in the model. It was connected to the foot through three joints. Each of these joints has its own characteristics determining the interactions of the foot with the shoe and the sole. Therefore, the kinematic chain of the lower limb model ends with the shoe element.

This model design has two main advantages. The first one is that it maintains uniformity with the dummy limb model necessary for the verification and analysis of results. The second one is a low calculation cost.

The mass of the elements of the model results only from the volume and density of FE elements, so it is equal to the mass of the lower limb skeleton. To reflect the mass of the entire limb, including soft tissues, an additional mass was assigned to the relevant body elements forming a kinematic chain.

### Model Verification

Verification of the developed model is very difficult due to the high costs of field testing, drop-tower, or sled tests. Therefore, it was decided to use in the process the results published in the literature. Therefore, publications by Bailey et al. (2013), Bailey et al. (2015) were used, where the authors extensively describe the methodology and results as well as use a relatively large group of specimens; however, one may notice the fact that they were obtained from elderly donors and so may affect the results.

In the first stage, the H-III dummy with the Mil-LX limb was placed in the test bench model accordingly. The limb was positioned as in the experiment, keeping the 90° angle in the ankle and knee joint. The seat on which the dummy was located was modeled as rigid surfaces. Contact was made between the surfaces and individual H-III segments: torso and back—backrest and hips—seat. The floor was also modeled as a rigid surface and contact was made between the shoes and the floor. These contacts are described using the built-in function for contacts between MB and FE elements, where the characteristics of the SLAVE element are assigned. In these cases, the abovementioned elements constitute individual segments of the dummy.

The experiment used Velcro straps to place and press the dummy’s legs against the floor—the experiment was carried out in the horizontal plane. To reflect these conditions and to stabilize the contacts, a presimulation was carried out with a switched gravitational effect in the direction of the *z*-axis of the model, i.e., vertical dummy axis. Naturally, the gravitational interaction in the main simulation was identical to the experiment, i.e., the arrow axis of the dummy.

The impactor weighing 32.44 kg was modeled as a rigid FE element. The impactor was assigned to the trans joint that allows the object to move only in one direction; in this case, parallel to the *z*-axis, according to the movement of the impactor on the test bench.

The characteristics of the susceptible element that affects the impact of the impactor are unknown. Therefore, it was decided to control the accelerator impulse, which was selected on the basis of known parameters and results of the experiment. The first part of the impulse affects the speed at the moment of impact, and the second part of the impulse contains a delay resulting from a collision with the floor and braking by a flexible element.

The comparison of the parameters and results of the experiment with the simulation is shown in Table 1. The results were highly consistent under almost identical conditions, which is the basis for further verification of the model.

In the second stage, the terms of the experiment were subjected to a dummy model with the author’s model of the anatomical lower limb described above. The simulation conditions were identical to those in the first stage. The same dummy and leg position were maintained for both models: dummy with the Mil-LX limb and dummy with the anatomical limb.

For both limb models, the compressive force exceeded a safe value in relation to the injury criteria, which can lead to injury at the AIS2+ level, i.e., tibia fracture. The stress value in the tibia reaches 160 MPa @ 7 ms, thus exceeding the yield point.

### Experiment Scenarios

**FIGURE 3:**
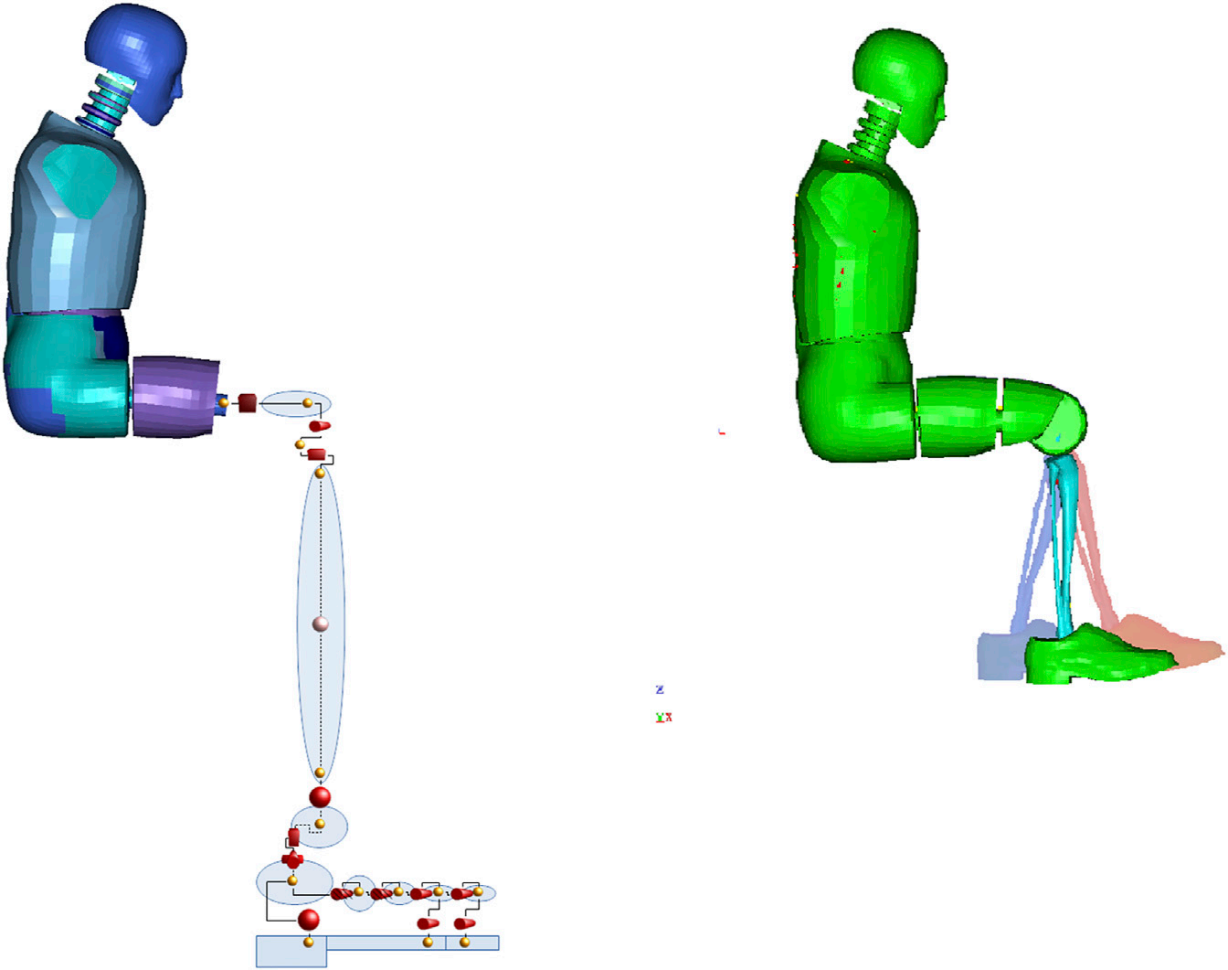
Model structure and model with geometry representation (visualization of positions 75° and 105°—blue, 90° and 90°, and 105° and 5°—red).

The first group of scenarios was conducted to analyze the impact of the angle in the knee and ankle joint on injuries. The experiment was conducted in the configuration described in Bailey et al., but with the anatomical lower leg model and with an acceleration impulse increased to correspond to the explosion of 10 kg of TNT under the center of the vehicle (level 4 of anti-mine protection). Load conditions were introduced to model as displacement vs. time assigned to baseplate under feet (Gzik et al., 2017). Seven scenarios were prepared including a reference position of the limb (90° and 90°). Three positions were set by increasing knee flexion while increasing dorsiflexion of the foot by 5°, 10°, and 15°, respectively, and three that assumed increasing knee extension relative to the base position with plantar flexion also by 5°, 10°, and 15°. The height of the foot support was determined for each case. The reference and extreme positions are shown in Figure 3. The summary of the results obtained is presented in Table 3.

**TABLE 3:** Comparison of the experiment with the simulation results.

Position	Peak stress value (MPa)			Force in ankle joint (kN)	
	Tibia	Talus	Calcaneus	Compression force (F_z_)	Shear force (F_x_)
75° and 105°	160	146	154	3.18	5.51
80° and 100°	160	132	151	3.63	7.23
85° and 95°	160	130	121	5.43	12.08
90° and 90°	160	140	145	4.85	11.13
95° and 85°	160	147	138	3.02	10.36
100° and 80°	160	160	160	2.35	10.54
105° and 75°	160	137	158	1.39	13.06

The second group of scenarios was a case study originating from a multi-variant analysis presented by Gzik et al. (2017), Gzik et al. (2016). Out of 162 cases, three were selected for closer inspection. The soldier was placed sitting on the left-hand side, in a seat that was the closest to the vehicle center in the troops compartment of the Rosomak APC. Geometry of hull, compartment, and seat was modeled basing on technical data and measurement of real object. Parameters of the vehicle floor kinematics (the table of displacement vs. time) corresponding to explosion of 10 kg of TNT under the front axle, rear axle, and compartment were used as loading conditions.

## Results

### Impact of Knee and Ankle Joint Angle

In each position, stress in the tibia reached a limit of 160 MPa. Only in position 100° and 80° did the stress in the talus and calcaneus reach 160 MPa. The highest compression force in the ankle joint was registered in position 85° and 95°, and the lowest in position 105° and 75°. Obtained values are summarized in Table 3.

### Case Study

In the case of explosion under the front axle, which, theoretically, is the least dangerous because it is distant from the landing compartment, the maximum value of stress in the tibia was 83 MPa. In the remaining cases, the stress in the tibia reached the limit value of 160 MPa. The stress value in the talus and calcaneus did not reach in any case an alarming level and was 33, 120, and 124 MPa, respectively.

## Discussion

**FIGURE 4:**
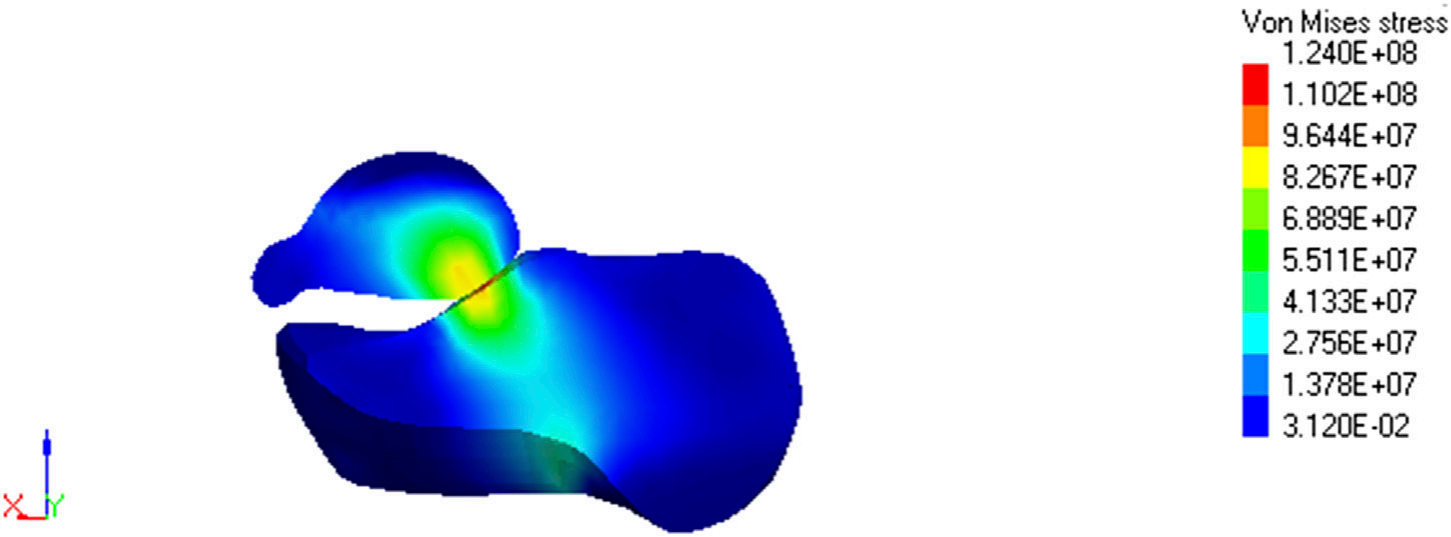
Stress field maps of tallus and calcaneus (ankle joint) at 105° and 75° position.

All seven limb settings reached a stress limit of 160 MPa. However, there are differences in the development of stress fields depending on the position. At position 90° and 90°, the stress concentration is in the shape of a helix starting halfway along the bone length, and the highest concentration occurs in the anterior part of the tibia. As the angle in the knee joint increases, the stress fields separate to form concentration in the upper-posterior part of the bone and a larger, main concentration field in the anterior-lower part of the bone. Reduction of the angle at the knee leads to the unification of the high stress value on the helix surface (Figure 4). The observed shape of the stress fields suggests various mechanisms of possible fracture of the tibia. In the case of a larger angle in the knee joint, stress fields located mainly in the anterior part of the bone indicate a flexural fracture mechanism. In contrast, the concentration of stress circulating around the entire bone circumference may indicate a compression or shear fracture mechanism (Figure 4).

**FIGURE 5:**
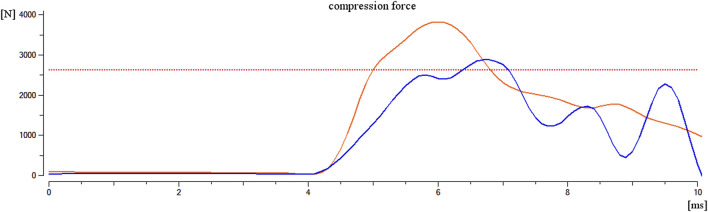
Comparison of compressive force in the lower tibia for the Mil-LX model (orange line) with compressive force in the ankle for the anatomical limb model (blue line); the red line indicates the 2.6-kN injury criterion.

Stress values in the calcaneus and talus bones are greater when the knee extension is increased. The stress concentration is from the bottom of the calcaneus towards the lower ankle joint and further from this place through the ankle bone towards the upper part of the ankle joint. The reduced knee extension results in greater stress dissipation in these bones (Figure 5).

This evolution of stress in the bones of the ankle can be explained by the fact that in the case of stronger bending of the knee and a simultaneous attachment of the back of the foot, the front part of the longitudinal arch of the foot takes a greater part in receiving the load from the floor direction, and thus the ankle is loaded from two directions: heel to floor contact and indirectly from the midfoot. Similar mechanisms were observed by Wong et al. (2016).

**FIGURE 6:**
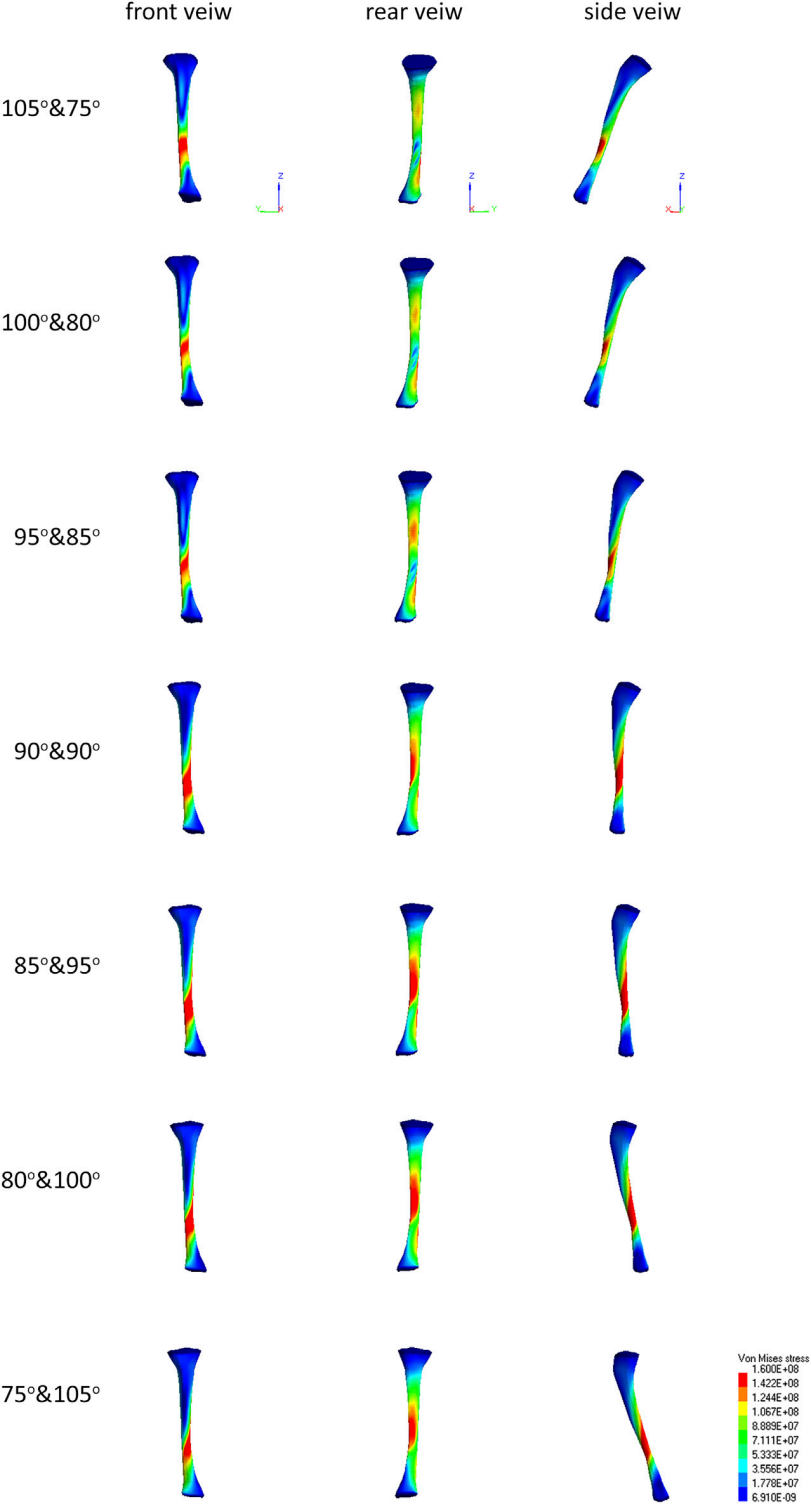
Stress field maps of tibia for different positions.

There are injury mechanisms characteristic of the effects of explosions, e.g., fracture of the calcaneus deck-slap (Ramasay et al., 2011b). It is not possible to directly link injuries, and more specifically fractures, which affected soldiers in combat conditions with the results of the simulation due to the lack of accurate information about the conditions where the injury occurred. Among the data collected during numerical tests, it was observed that stress fields could indicate fractures similar to those that actually occurred in the event of an explosion under the vehicle. The similarities described mainly apply to the calcaneus and tibia. In particular, concentration fields of stress in position 105°and 75° show similarity to deck-slap fracture of calcaneus (Figure 6).

The analysis of 162 cases using the Mil-LX model presented by Gzik et al. (2017) indicated that the location of the explosion under the vehicle is important for the risk of injury. The analysis of the selected three cases using an anatomical model showed that there were no significant differences in the mechanism of limb loading, whereas the stress maps obtained changed in proportion to the compressive force in the tibia. This suggests that bringing small components, next to the main component, into the acceleration pulse does not have a significant impact on the risk of injury in the case of the lower limb. However, the amplitude of the said pulse is much more important.

The position of the limb has a much greater impact on the risk of injury and even its type. This fact was demonstrated by studies carried out using an anatomical model where seven configurations of angles in the joints were analyzed. Two different mechanisms of injury have been observed. An increase in the angle in the knee joint (extension) causes a change in the mechanism of injury of the tibia from a compressive mechanism to flexing mechanism, while changing the mechanism of loading the ankle.

**FIGURE 7:**
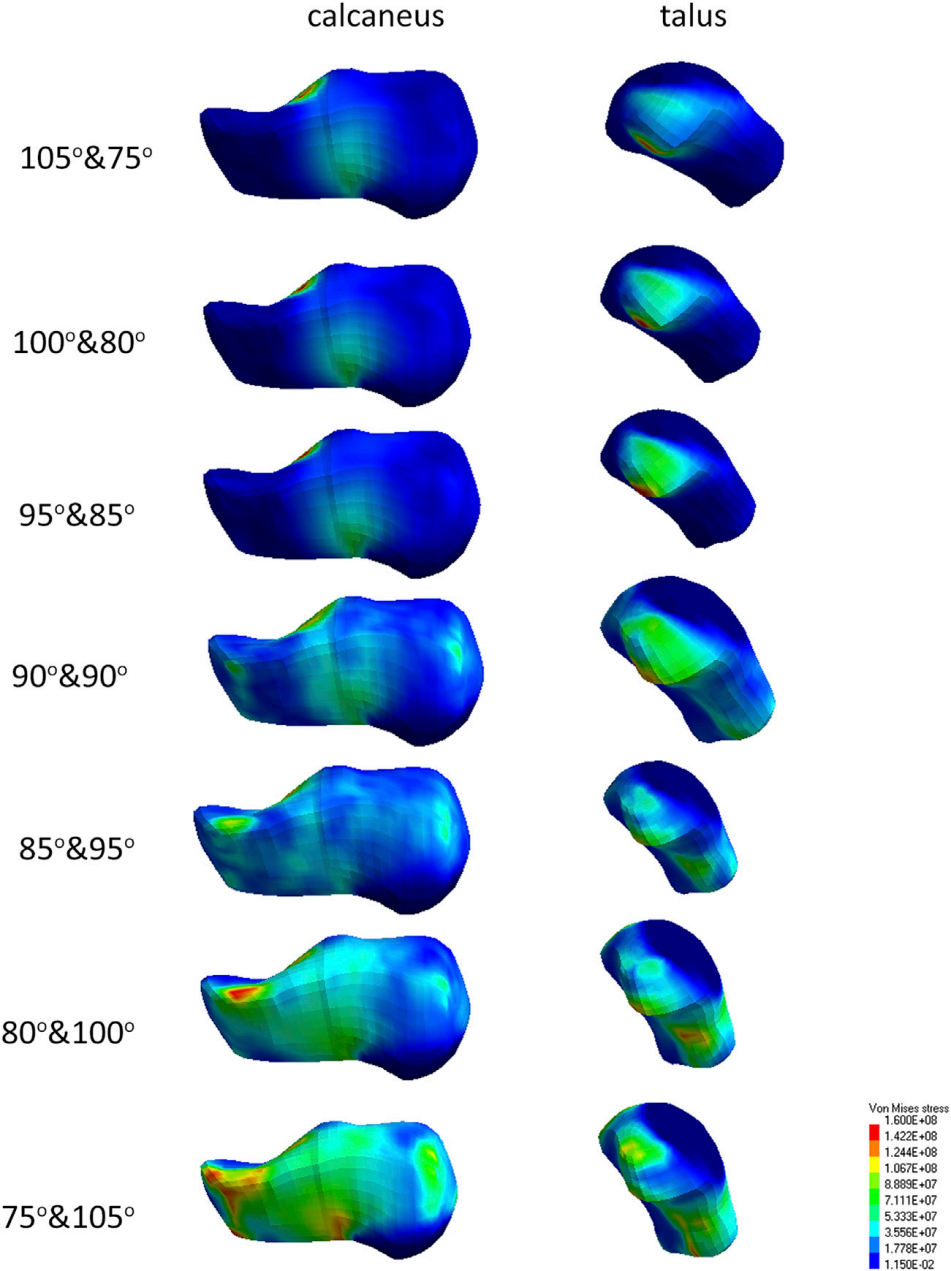
Stress field maps of tallus and calcaneus for different positions.

Verification of model was made based on an *in vivo* experiment described in literature (Bailey et al., 2013, 2015) and offers satisfactory consistency of results in condition described in literature (Table 4). An anatomical model was also compared to an already verified model of Mil-LX lower leg, and results are shown in Figure 7. The first group of seven scenarios with different leg positioning was conducted in configuration as during the verification process but with loading conditions taken from a previous work done within the research project. The second group of three scenarios was conducted with configuration as in real-life environment, which is the interior of the Rosomak APC model validated in previous work (Gzik et al., 2017, 2016). Verification with use of literature data was necessary due to difficulty of *in vivo* blast experiments including cadavers. The first step of verification is consistent. The transition from loading conditions used by Bailey et al. to conditions corresponding to 10 kg of TNT may be debatable; however, comparison of response of the anatomical model with the Mil-LX model (well-verified model delivered by Madymo environment) allows the assumption of positive validation.

**TABLE 4:** Obtained results: peak stress values of bones and forces in ankle joint.

	Bailey et al. (2013), Bailey et al. (2015)	Numerical simulation
Impactor velocity in moment of impact (m/s)	8.09	7.7
Max. foot acceleration (m/s^2^) @ (ms)	2727 @ 2.11	2972 @ 2.96
Lower leg compression force (kN) @ (ms)	3.77 @ 5.50	3.82 @ 5.95

The research presented in the current article contributes to the extension of knowledge about the biomechanics of the soldier’s lower limbs in the event of an explosion under the vehicle. The research was conducted in the light of the latest research methodology and the latest publications. The developed methodology and numerical models can be successfully used in other studies in this area.

## Data Availability

The datasets presented in this article are not readily available because due to military characteristic of project “Afgan” data is partially non-public. Requests to access the datasets should be directed to slawomir.suchon@polsl.pl.
