# Supplementary material for: Lower Leg Injury Mechanism Investigation During an IED Blast Under a Vehicle Using an Anatomic Leg Model
Source: Front Bioeng Biotechnol. 2021 Nov 17;9:725006. doi: 10.3389/fbioe.2021.725006 (PMC8635724; doi:10.3389/fbioe.2021.725006)
Supplement: Supplementary file 1 [file Table1.docx]

# Supplementary Material

Table 1. Hexagonal mesh parameters for the most important model elements

| **Mesh parameter** | | **Off-limit elements** | **Worst element** |
| --- | --- | --- | --- |
| **Jacobian**  **≥0.5** | tibia | 0% | 0.51 |
|  | fibula | 3% | 0.37 |
|  | talus | 2% | 0.37 |
|  | calcaneus | <1% | 0.25 |
|  | total | <1% | 0.25 |
| **Wrapage**  **≤ 10** | tibia | 1% | 42.68 |
|  | fibula | 4% | 55.32 |
|  | talus | 12% | 80.27 |
|  | calcaneus | 5% | 61.24 |
|  | total | 2% | 80.27 |
| **Aspect**  **≤5** | tibia | <1% | 5.19 |
|  | fibula | 0% | 5.75 |
|  | talus | <1% | 5.07 |
|  | calcaneus | <1% | 6.08 |
|  | total | <1% | 6.08 |
| **Skew**  **≤60** | tibia | <1% | 65 |
|  | fibula | <1% | 74 |
|  | talus | 3% | 80 |
|  | calcaneus | 2% | 80 |
|  | total | <1% | 80 |
